# Plant Viruses Infecting *Solanaceae* Family Members in the Cultivated and Wild Environments: A Review

**DOI:** 10.3390/plants9050667

**Published:** 2020-05-25

**Authors:** Richard Hančinský, Daniel Mihálik, Michaela Mrkvová, Thierry Candresse, Miroslav Glasa

**Affiliations:** 1 Faculty of Natural Sciences, University of Ss. Cyril and Methodius, Nám. J. Herdu 2, 91701 Trnava, Slovakia; 2795151@student.ucm.sk (R.H.); daniel.mihalik@ucm.sk (D.M.); michaela.mrkvova@ucm.sk (M.M.); 2 Institute of High Mountain Biology, University of Žilina, Univerzitná 8215/1, 01026 Žilina, Slovakia; 3 National Agricultural and Food Centre, Research Institute of Plant Production, Bratislavská cesta 122, 92168 Piešťany, Slovakia; 4 INRAE, University Bordeaux, UMR BFP, 33140 Villenave d’Ornon, France; thierry.candresse@inrae.fr; 5 Biomedical Research Center of the Slovak Academy of Sciences, Institute of Virology, Dúbravská cesta 9, 84505 Bratislava, Slovakia

**Keywords:** plant viruses, *Solanaceae*, agro-ecological interface

## Abstract

Plant viruses infecting crop species are causing long-lasting economic losses and are endangering food security worldwide. Ongoing events, such as climate change, changes in agricultural practices, globalization of markets or changes in plant virus vector populations, are affecting plant virus life cycles. Because farmer’s fields are part of the larger environment, the role of wild plant species in plant virus life cycles can provide information about underlying processes during virus transmission and spread. This review focuses on the *Solanaceae* family, which contains thousands of species growing all around the world, including crop species, wild flora and model plants for genetic research. In a first part, we analyze various viruses infecting *Solanaceae* plants across the agro-ecological interface, emphasizing the important role of virus interactions between the cultivated and wild zones as global changes affect these environments on both local and global scales. To cope with these changes, it is necessary to adjust prophylactic protection measures and diagnostic methods. As illustrated in the second part, a complex virus research at the landscape level is necessary to obtain relevant data, which could be overwhelming. Based on evidence from previous studies we conclude that *Solanaceae* plant communities can be targeted to address complete life cycles of viruses with different life strategies within the agro-ecological interface. Data obtained from such research could then be used to improve plant protection methods by taking into consideration environmental factors that are impacting the life cycles of plant viruses.

## 1. Solanaceous Plants as Host of Viral Pathogens

The *Solanaceae* family is a monophyletic dicot group, which contains widely cultivated crops with individual species serving as a food source, as a source of bioactive molecules or as ornamentals [1]. Species belonging to this family, such as the potato (*Solanum tuberosum*), tomato (*S. lycopersicum*), pepper (*Caspicum annuum*) or tobacco (*Nicotiana tabacum*), are grown on all continents with temperate or tropical climates and are commonly found in many households worldwide [2]. Beside crops, medicinal plants used for alkaloids production can be found in the same family, e.g., deadly nightshade (*Atropa belladonna*), black henbane (*Hyoscyamus niger*) and jimson weed (*Datura stramonium*). Plants from the *Solanaceae* family have also played an important part in genetic research over the last hundred years [1]. Cultivated *Solanaceae* species often grow side by side within the same ecosystem with wild species of the family, some of which are frequent weeds [3]. Under all complex cultivation contexts, *Solanaceae* plants are exposed to infectious pathogens, including viruses.

Viruses are small infectious agents that are capable of replication only within cells of living organisms. The first virus recorded, tobacco mosaic virus (TMV), was discovered in tobacco, a *Solanaceae* [4]. Moreover, TMV has been acknowledged as a preferred didactic and symbolic model to illuminate the essential features that define a virus [5]. According to the International committee on taxonomy of viruses (ICTV), 6 orders, 32 families and 141 genera, comprising 1901 plant virus species, are currently recognized [6]. This number is currently rapidly increasing thanks to the application of high throughput sequencing technologies enabling an unbiased robust analysis of plant viromes [7,8], leading to the discovery of a wealth of new viruses (e.g., [9,10]).

Means of transmission from one host to another vary among viral species. The transmission can be vertical or horizontal, the latter being frequently mediated by vectors or by contact. Viruses infecting solanaceous species are mostly transmitted by insect vectors, in particular aphids [11,12]. Other vectors are also capable of transmitting some viruses, as thrips mediating the transmission of tospoviruses [13], whiteflies (e.g., *Trialeurodes vaporariorum*, *T. abutilonea*, *Bemisia tabaci*) of torradoviruses [14,15] or nematodes as seen with tobacco rattle virus (TRV) [16]. Transmission by soil Chytridiomycetes or Plasmodiophorids [17] is known for tobacco necrosis virus (TNV) as zoospores of *Olpidium brassicae* [18], or for potato mot top virus (PMTV) as those of *Spongospora subterranea* [19]. A similar transmission route has also been hypothesized (but so far not confirmed) for pepino mosaic virus (PepMV) and *Olpidium virulentus* in tomato plants [20], and the existence of an additional transmission pathway for potato virus Y (PVY) involving mycorrhizal networks created by hyphae of arbuscular mycorrhizal fungi has been hypothesized [21], again without confirmation so far. On the other hand, vertical transmissions through seeds or pollen are also documented, e.g., tobamoviruses [22], PepMV [23] or tomato torrado virus (ToTV) [24] infecting various *Solanaceae* species.

Viral phytopathogens present a great risk for plant production in agriculture as some of them can induce severe diseases [25]. According to Anderson et al. [26] viruses cause about half of emergent infectious diseases in plants. The majority of these emergence events are caused by the introduction of a pathogen (71%), followed by changes in vector populations as a second cause (17%). Recombination events of mutant strains accounted for 5% of emergence events in this study. In *Solanaceae* species, previously documented emerging viruses, such as ToTV, tomato chlorosis virus (ToCV), tomato infectious chlorosis virus (TICV), PepMV or tomato brown rugose fruit virus (ToBRFV), all of which have been reported to infect tomato plants in Europe, have caused significant yield and economical losses [27,28].

## 2. Impact of Global Environmental Changes on Plant Virus Fitness

The introduction of pathogens to a new geographic area as a cause of emergence is generally linked to the ongoing global changes. The globalization of markets and the novel ways of quickly transporting goods provide an opportunity for long distance dispersal of pathogens [29]. The recent rapid dispersion of ToBRFV to several continents provides a good illustration of such trends [30,31]. Another illustration of this trend is the report by Just et al. [32] of the presence of tomato yellow leaf curl virus (TYLCV) in imported packaged tomatoes in Sweden and Estonia. Another factor influencing virus emergence and spread is climate change [33]. With the rise of annual mean temperatures, changes in vectors distribution are already seen and can be further expected, providing novel opportunities for viral spread.

With shifting climate conditions and changes in agricultural practices and land use, it is also expected that new areas will become used for agricultural purposes providing novel opportunities for contact between crops and native flora, or opportunity for invasive weed species to spread to formerly cold/temperate areas [34]. As plant species are expected to spread to new areas, virus emergence can work in two ways: native plants can serve as new hosts for crop-infecting viruses, or novel crops can be infected by viruses formerly present in the native flora [35,36]. Both of these phenomena can be expected to happen simultaneously in the environment. The changing climate can also induce changes in agricultural practices that can be expected to have an impact on viral emergence events [37].

## 3. Covering All Infection Pathways by Taking into Account the Agro-Ecological Interface

Crops are usually grown within a complex agro-ecosystem. The term agro-ecological interface is used to describe the border between cultivated and wild plant communities [38]. As viruses can infect both crop and wild plant species and can be dispersed by various vectors colonizing both types of species, it is possible to observe the movement of viruses between these two environments. A number of factors differ between these environments and are expected to impact viral populations and the development of epidemics (Table 1), emphasizing the need for research considering the agro-ecological interface as a whole. The importance of interactions at the agro-ecological interface is underlined by the fact that wild plants can serve as reservoirs of crop-infecting viruses [39] and, conversely, that wild species growing close to crops can be infected by crop viruses in a spillover process [35]. By taking into account the agro-ecological interface, it is, therefore, possible to keep track of interactions between and within groups of plants. It is also apparent from previous studies that wild plants can pose as refugium for viruses during intercropping periods; most solanaceous crops are annuals that are not grown all year long (at least in unprotected cultivation in temperate regions) and wild perennial plants may thus serve as virus reservoirs during the inter-crop season. It was shown, for example, that a wild perennial, black nightshade (*Solanum nigrum*), can serve as a TYLCV reservoir [40].

Most studies of plant viruses are focused on crop species and on crop-virus interactions. There are much fewer studies addressing the effects of viruses on wild plants. When it comes to wild species, most studies are concerned with the directional spread of viruses from wild to crop species. Fewer address the transmission of viral infection from cultivated to wild plants. Overall, we are also lacking much knowledge about the natural virus/plant interactions within the wild compartment [50], even though there are indications that natural life cycles of plant viruses in their wild host populations are the result of a co-evolution process lasting millions of years [51,52]. As agro-industrial practices have been very widely used in recent history, the natural plant-virus interactions within crop fields can be expected to have been substantially modified due to human induced global changes and the confounding factors associated with them [26]. In this respect, it is remarkable that datation efforts have suggested that the explosive radiation that has made the potyviruses the largest genus of plant-infecting RNA viruses might be associated with the development of early agriculture 6600 years ago [53].

As plant viruses are usually looked upon only from an agricultural perspective, plant viruses are mostly considered as a source of economic losses, and thus, as having a negative impact. Economical losses caused by viral pathogens infecting solanaceous plants can be illustrated by tomato-infecting begomoviruses that are mentioned among the top ten list for economically important viruses [54], which reacted to a previous publication of the top 10 plant viruses in molecular plant pathology [55] where six out of ten viruses are infecting solanaceous hosts. Other cases include potato virus X (PVX), potato leafroll virus (PLRV) and PVY viruses infecting potato plants, causing economical losses worth 5.5 million US dollars in the UK [56], or TICV infecting tomato crops in California with estimated economical loss of 2 million US dollars [27]. This view may however be skewed, as the natural life cycles of plants and viruses are undoubtedly firmly connected by the obligate parasite-host relation between them. The ecological view on plant viruses, even though complex and still lacking sufficient data, can provide precious information for the fields of virology and epidemiology. These are also crucial information for the prevention of future epidemics, and sustainability of food security. The necessity to take into account the viruses present in wild plant populations has thus become more apparent, as we cannot expect to fully understand virus natural life cycles by only targeting cultivated crops [42], since viral cycles can be impacted by human alterations of numerous environmental variables as a consequence of agricultural practices aiming to increase yield. It is also becoming apparent that the extensive crop monocultures, lacking genetic heterogeneity, contribute to the emergence of plant viruses [41]. In wild ecosystems, plant viruses are subjected to more diverse and possibly stronger selection pressures, as a consequence of biotic and abiotic stress, more limited availability of suitable hosts and vectors or even of competition between viruses [57]. At the same time, there are documented cases of crop-infecting viruses undergoing recombination in wild native plants, a clear demonstration of the potential role of wild species in the evolution of crop-infecting viruses. A good example of this situation concerns tomato yellow leaf curl disease (TYLCD) and black nightshade, which was found to harbor mixed begomovirus infections more often, providing optimal conditions for the development of well adapted recombinants [58]. At the other extreme, it has been suggested that under some conditions, it could be beneficial for a virus to preserve, or even improve its host plants fitness in order to increase its chances of successful transmission [59]. The virus dependence on plant populations is quite clear, as viruses can only replicate within host cells, but as mentioned previously, recent studies have highlighted some potentially positive effects of viruses on plants. For example, Xu et al. [60] reported increased drought tolerance in a number of plant species as a consequence of infection by several plant viruses, including tomato plants infected by cucumber mosaic virus (CMV). These findings hint at the broad spectrum of plant virus functioning and of their ecological impact in a broader and hopefully clearer way.

The information about natural plant-virus interactions is crucial for the future of agricultural production and of food safety, by informing the prediction of future emergence events or of future epidemies and by supporting the deployment of appropriate control measures. One recently highlighted facet of these interactions is the realization that there might exist, in some cases, a positive effect of mixed virus infection in wild plants [61,62], and possibly also in cultivated ones [63]. Mild viruses are currently being used to control virus-induced diseases, by pre-inoculating plants with an attenuated or a symptomless variant, and by applying the knowledge of natural virus competition during super-infection for plant protection purposes [45]. Examples on *Solanaceae* plants are numerous [64], including the use of a mild isolate of ToMV Fukushima strain (L11A) to protect tomatoes against highly virulent ToMV isolates [65], a mutant PepMV KD [66] or a mild isolate of the CH2 strain [67,68,69] to protect them from secondary infection by wild-type PepMV. The term “antagonism” is used to describe a type of virus-virus interaction in mixed infection, where infection by one virus prevents infection, or suppress symptoms, accumulation or transmission rates of another one. As indicated above, this type of interaction is widely used for protecting plants by the means of cross-protection [69]. The importance of virus infection timescale can be crucial for plants. Regarding wild solanaceous plants, Grupa & Syller [70] presented the first example of potato virus M (PVM) cross protection with jimsonweed as a host plant. Cross protection was aimed against the PVM Uran strain by pre-inoculation with the PVM I-38 mild strain. Other means of plant protection are being used as well. Solanaceous crops are being bred for resistance against viral pathogens, as can be observed on tomatoes carrying the Tm-22 gene of resistance against ToMV [71]. The down-coming of genetic resistance is in the ability of viruses to adopt in the form of resistance-breaking isolates, as reported for Tm-22 breaking isolates of ToMV [72,73]. New methods for solanaceous plant protection are still being developed, such as dsRNA external treatment of plants triggering RNA interference, inhibiting TMV virus propagation [74].

The previous limited interest given to wild plant virus research can be exemplified by the studies of henbane mosaic virus (HMV, Potyvirus, family Potyviridae). HMV was first described from black henbane plants [75]. Later on, HMV was found to be infecting deadly nightshade, *Physalis alkekengi* and jimson weed [76]. Even though all the main hosts of HMV are members of the *Solanaceae* family, research on this virus has been scarce, and it was only recently that HMV was found naturally infecting field-grown tomato plants in Slovenia [77]. In this case, HMV was a part of mixed infection with PVM (carlavirus) and southern tomato virus (STV). The Slovenian HMV isolate (termed HMV-SI/L) was further found in the same study to be able to infect a range of tomato cultivars, a wild tobacco species (*Nicotiana benthamiana*) and wild *Solanaceae* species (black henbane, jimson weed).

## 4. Narrowing Down Virus Life Strategies to *Solanaceae* Family Phylogenetic Level

There are indications suggesting that closely related species are more likely to exchange viruses with the wild landscape setting of a reservoir, or by being back infected from crops. The host diversity of 480 plant viruses was previously evaluated [78]. Significantly stronger barriers to infection were documented for viruses as the host diversity range crossed the taxonomic family border, with a continuous decrease in host diversity observed in higher taxonomy ranks. By further analysis of the plant-virus infectivity matrix of 37 plant viruses and 28 plant species, significant modularity was detected by these authors, where each module was associated with a plant family. In other words, this analysis implies that phylogenetic distance between prevalent and susceptible host plants can be linked to the likelihood of viral emergence. One module identified in this study was associated with the *Solanaceae* family, suggesting that studies limited to *Solanaceae* plants can be relevant and informative, while being convenient for study design and sampling [78]. This notion is supported by a study of ToCV, where four wild plants from different families were evaluated as virus sources in transmission experiments. Black nightshade was proven to be the most efficient source for virus transmission of a ToCV isolate from tomato. Thus, both the source plant of the ToCV isolate used, and the most efficient transmission source species, belong to the *Solanaceae* family [79].

It is, thus, possible to simplify a vast number of ecological factors by limiting the sampling to a selected plant family, and still include most of virus life strategies and plant-virus interactions [78]. The potential of including most virus strategies is very hopeful, as viruses and viroids from more than 40 genera are naturally infecting solanaceous plants (Table 2). The *Solanaceae* plant family, with its major interest for agriculture, virology and epidemiology studies, is a good candidate for such host family-centered studies. Including cultivated and wild plant viruses and the interactions between and within these groups of plants will undoubtedly provide more valuable information for the prevention of future epidemics, as the virus emergence process involves multiple species in communities on a landscape level [80]. It is suspected that plant viruses may be experiencing higher or more complex selection pressures in wild plant populations because of the increased within-species genetic heterogeneity compared to the limited genetic heterogeneity seen in crop plant populations [43]. At the same time, given the frequent occurrence of both cultivated and wild plants from this family in most parts of the world, research conducted on solanaceous plants could cover a wide spectrum of virus life cycles and their interactions.

In order to perform a viral ecology study focusing only on members of the *Solanaceae* family, several factors need to be considered. In order to cover the agro-ecological interface in its entirety, it is indispensable to include both wild and cultivated plant species. This ensures that all virus infection pathways can be at least partially included, thus providing data that may help to evaluate their epidemiological impact. It is also necessary to consider the consequences of long-term agricultural practices on plant virus populations, including a weed species, which is taxonomically closely connected to crop species can be of great importance, as it may shed light on the underlying processes during virus transmission, which was proven to be often connected with phylogenetic distance [78]. The vast amount of research done on viruses infecting model plants from the *Solanaceae* [1] is, thus, a great resource on which it is possible to build further.

Interesting experiments involving two tospoviruses, namely tomato spotted wilt virus (TSWV) and iris yellow spot virus (IYSV), infecting jimson weed plants was conducted by Bag et al. [85]. This provided the first report of synergism between ambisense viruses in wild *Solanaceae* plants. While jimson weed is infected by TSWV in a systemic manner, it restricts the systemic movement of IYSV, which is limited to the inoculated leaves and fails to move to younger ones. In mixed infection, symptom severity increased as compared to single TSWV infection, and IYSV could be detected in uninoculated leaves. These results suggest that virus synergism within wild *Solanaceae* plants can lead, in some cases, to resistance-breaking or host range expansion as observed before on cultivated *Solanaceae* [86]. Examples of wild *Solanaceae* plants roles in virus life cycles can be observed in the case of begomoviruses causing TYLCD, which is known to act as bundles of genomes undergoing mutation and recombination, called quasispecies. When Sánchez-Campos et al. [87] compared four plant hosts infected by three viruses causing TYLCD, the black nightshade wild reservoir plant had higher quasispecies heterogeneity than tomato, showing its potential to contribute to viral evolution by providing a larger supply of variants for the natural selection processes.

A recent study of the emerging tomato leaf curl New Delhi begomovirus (ToLCNDV) in Spain [88] also underlined the importance of wild *Solanaceae* plants in research regarding virus population dynamics. It was found that ToLCNDV shows higher within-host genetic diversity and has a higher mutation rate in jimson weed compared to cultivated plants. Further, because of jimson weed extensive presence in cucurbit fields, it can serve as an inoculum source, as confirmed by the absence of signs of segregation in the ToLCNDV population, even when samples were collected from host plants belonging to different families. Another study by Ma et al. [89] took a closer look at the viral populations exchange between tomato and black nightshade. A novel virus called solanum nigrum ilarvirus 1 (SnIV 1) was capable of infecting both black nightshade and tomato, but was only prevalent in black nightshade populations. SnIV 1 was found in black nightshade regardless of whether plants grew side by side with tomato or not, suggesting that while SnIV1 is able to remain in black nightshade populations independent of the presence of tomato, infection in tomato was a consequence of the SnIV 1 transfer from black nightshade. This novel virus is phylogenetically close to other ilaviruses known to cause diseases in tomato, such as tomato necrotic spot virus (ToNSV) and tomato isolates of parietaria mottle virus (PMoV), which are responsible for outbreaks in the USA and Europe, respectively [90]. This suggests a scenario of occasional, possibly pollen-mediated, transfer from a black nightshade reservoir to neighboring tomato crops followed by further spread from tomato to tomato. At the same time, there seems to be an inability to persist in tomato over the long term, possibly as a consequence of limited or nil cultivation during the winter period. Further, it was found that PVY infection was prevalent in black nightshade populations only at tomato sites, which suggests a PVY spillover effect from tomato, opposite to the SnIV-1 example. In the same study, broad bean wilt virus (BBWV) was detected in black nightshade plants only, even though its ability to infect tomatoes has been previously demonstrated [91], suggesting the existence of (an) unknown ecological barrier(s) preventing BBWV1 efficient spread from black nightshade to tomato under local conditions. It is apparent that viral exchange between cultivated and wild plants is a relevant factor for the functioning of the populations of these three viruses, and that transfer can happen in both directions, from wild to cultivated and from cultivated to wild, driven by underlying biological and ecological forces that need to be better understood in the future for more effective prevention practices [89].

In a study addressing virus flow from wild plant to crops within the *Solanaceae* family, yellow tailflower mild mottle virus (YTMMV, Tobamovirus) was isolated from an indigenous wild host plant, and serially passaged through three exotic host species. Within the YTMMV genome, six polymorphic sites were detected during the experiment. One of these mutations was a non-synonymous one, observed only when YTMMV was passaged through tomato. The mutant increased in titer during tomato passages, and after passaging, it had higher reproductive fitness in tomato compared to the original isolate [43]. From this experiment, it is obvious that host changes and the ensuing adaptation to the novel host may play an important role in the evolutionary dynamics of viruses, including those infecting *Solanaceae*. Adaptations improving virus fitness in different hosts are indeed known to be key in the emergence of viruses by host change [92]. A study of weeds growing within or close to potato fields as a potential PLRV inoculum source [3] tested 26 different weed species for PLRV presence by ELISA, using RT-PCR for confirmation. Six of these species were found naturally infected by PLRV, of which four were from the *Solanaceae* family (*D. stramonium, Physalis minima, S. nigrum, Withania somnifera*). The role of wild Solanaceous plants as a potential source of virus infection was addressed and confirmed for various virus/plant combinations in a number of other studies [93,94]. However, caution should also be exercised, in particular when virus presence is only ascertained by serology and not backed up by molecular data, demonstrating that the same viral isolates are indeed shared between the wild and cultivated hosts. Such data are indeed absolutely needed in order to exclude the hypothesis of different viral strains or ecotypes independently circulating in the wild and cultivated host plant populations.

## 5. Conclusions

One of the main plant virus research objectives aims to control virus spread on crop plant communities in order to reduce yield or quality losses. In the specific case of solanaceous crops, great economic losses caused by virus infection have been documented [95]. Because viral infections in plants cannot be cured, it is important to identify the presence of virus (es) in infected plants by timely and effective diagnostic methods, and to prevent further virus spread by eradicating infected plants or, in the best case, by interrupting the contamination pathway. Indeed, due to a lack of curative treatment measures, prophylactic control measures, consisting of a combination of vector management, biosecurity measures and cultural practices, constitute a major pillar of plant virus disease control strategies [45]. Techniques such as meristem cultures, chemo-, thermo-, electro- and cryotherapy are widely used, individually or in combination, for the production of virus-free plants, such as for potato, which is almost exclusively propagated vegetatively through tubers [96]. Even such virus-free plants can later be infected by viruses under field conditions, as described throughout this review. Some plants of agricultural importance were bred to have resistance against selected virus pathogens. However, as in many other examples, the study of García-Andrés et al. [97] pointed out, in the *Solanaceae*, the possible contribution of the deployment of the resistance gene Ty-1 in tomato in the emergence of resistance breaking TYLCV variants. Other resistance genes in tomato have also been reported to be broken when plants were infected by new viral strains. Such cases include, for example, Tm-22 resistance breaking by the emerging ToBRFV [30], or Sw-5 resistance-breaking isolates of TSWV [98].

In conclusion, solanaceous plants continue to be a very interesting model for virology research because of their versatility, ubiquity and of the large amount of information available for virology studies. Moreover, due to their high susceptibility to a wide variety of plant-pathogenic viruses, several well-known model hosts in plant virology belongs to the *Solanaceae* family, e.g., *Nicotiana tabacum* or *N. benthamiana* [1,99]. We are aware of the fact that research focused on *Solanaceae* plants only will surely exclude some information on viruses from the available pool in the environment. Specialist viruses, which are not capable of infecting plants from this family, would not be detected at all, and generalist viruses could be underrepresented in number of hosts and infection pathways. Still, the *Solanaceae* family includes cultivated, wild, annual and perennial plants growing in various environments with different levels of intraspecific genetic variation and a variety of biological connections among individual species (Table 1). Therefore, their study throughout the agro-ecological interface should be able to cover a majority of plant virus life strategies. We believe that focusing on this plant family would mean a possible simplification of sampling and data management associated with environmental effects. Because of the signs of co-evolution, the small phylogenetic distance between plants, processes happening during virus transmission and spillback could be, thus, characterized more closely, providing information necessary for the adjustment of plant protection practices in a continuously changing environment.

## Figures and Tables

**Table 1:** Comparison of cultivated and unmanaged environments, focusing solely on solanaceous plants and viral pathogens infecting them.

Factors	Environments		Effects on Virus Populations
	Cultivated	Unmanaged	
Biodiversity of plant species	single or few species within field	considerably larger	Low biodiversity can facilitate epidemic development, in particular in the case of specialist viruses [41]
Genetic variability of individual plant species	very limited, often only a single cultivar	considerably larger	Low genetic diversity can facilitate epidemic development. It can also provide strong, unidirectional selective pressures driving, for example, the emergence of resistance-breaking virus isolates [42,43]
Chemical treatment (pesticides/insecticides)	common to rare	none	Chemical treatment can limit vector populations and therefore transmission rates for viruses transmitted by efficiently controlled vectors [44,45]
Period without vegetation (inter crop)	common	very rare, only caused by environmental factors (e.g., fires, floods)	Vegetation-free period disturbs virus life cycles. Only some viruses remain infective in soil/water for long period (e.g., Tomato mosaic virus (ToMV)). Otherwise, virus colonization of hosts will start anew [46,47]
Plant population life cycles	annual for many crops	annual/perennial	Perennial plants can serve as reservoirs for viruses outside the vegetation period in temperate climate [40]
Vegetation density	commonly dense, with uniform distances—(controllable)	ranging from sparse to very dense—(random)	Increased density of potential host plants can contribute to more efficient epidemics [43]
Environmental conditions (temperature, precipitation, humidity, wind)	commonly altered to optimal values in order to increase yield (watering, foliation, wind breakers, fertilizers, etc.)—(controllable)	dependent on weather and other environment conditions alone—(random)	Efficient plant growth may drive the development of large vector populations, thus contributing to more efficient epidemics. Extreme weather conditions such as strong winds and heavy rainfall wounding plant tissue can help transmission of contact-transmitted viruses by leaf-fall and rain splash [33,48]
Biodiversity of vector population	dependent on the host biodiversity and environmental conditions	dependent on the host biodiversity and environmental conditions	Vector populations biodiversity is expected to decrease together with plant populations diversity. Reduction of the populations of natural enemies of vectors may lead to higher vector populations and, eventually, to faster epidemics [49]
Origin of the plants	often introduced from other geographical areas	mostly local, but there can be invasive plant species present	Introduction of crops in new geographic areas may provide opportunities for novel host-virus encounters and drive the emergence of novel diseases [36]

**Table 2:** List of selected viruses naturally infecting plants of the *Solanaceae* family [81,82,83,84].

Virus Genera	Genome	Natural Transmission	Natural Solanaceous Hosts	Remarks
Virus Species				
**Alfamovirus**	**(+)ssRNA**			
AMV (alfalfa mosaic virus)		aphids/seed	pepper, tomato, potato, petunia, eggplant, sweet pepino, tamarillo	large host range
**Alphaendornavirus**	**dsRNA**			
BPEV (Bell pepper alphaendornavirus)		Seed/pollen	pepper, tomato, potato (Phujera)	
HpEV (Hot pepper alphaendornavirus)		Seed/pollen	pepper	recently described virus
**Alphanecrovirus**	**(+)ssRNA**			
PoNV (Potato necrosis virus)		*Olpidium brassicae*	potato	recently described virus
TNV-A (tobacco necrosis virus)		*Olpidium brassicae*	potato, tobacco	previously considered as one species with TNV-D (Betanecrovirus), a helper virus for Tobacco albetovirus 1, -2, -3
**Alphanucleorhabdovirus**	**(-)ssRNA**			
EMDV (Eggplant mottled dwarf nucleorhabdovirus)		leafhoppers	eggplant, tobacco, tomato, potato, pepper	
PhCMoV (Physostegia chlorotic mottle virus)		unknown	tomato	recently described virus
PYDV (Potato yellow dwarf virus)		leafhoppers	potato, pepper, tomato	
**Amalgavirus**	**dsRNA**			
STV (Southern tomato virus)		seed	tomato	
**Betanecrovirus**	**(+)ssRNA**			
TNV-D		*Olpidium brassicae*	tobacco	previously considered as one species with TNV-A (Alphanecrovirus)
**Betanucleorhabdovirus**	**(-)ssRNA**			
DYVV (Datura yellow vein nucleorhabovirus)		unknown	jimson weed, tomato	
**Begomovirus**	**ssDNA**			
More than 200 species in the genus begomovirs have been reported as naturally infecting *Solanaceae* species, e.g., ToLCNDV (Tomato leaf curl new delhi virus), TYLCV-Is (Tomato yellow leaf curl virus—Israel), TYLCSV (Tomato yellow leaf curl sardinia virus)		whitefly *Bemisia tabaci*	petunia, tomatillo, jimson weed, pepper, black nightshade, tobacco, tomato, eggplant	frequently large host range, often associated with alphasatellites (DNA-1) and betasatellites (DNA-β), many recently described viruses
**Carlavirus**	**(+)ssRNA**			
CPMMV (Cowpea mild mottle virus)		whitefly *Bemisia tabaci*	tomato, eggplant	
PotLV (Potato latent virus)		aphids	potato	
PVH (Potato virus H)		aphids	tomato, potato	recently described virus
PVM (Potato virus M)		aphids	tomato, potato, sweet pepino, bittersweet	
PVP (Potato virus P)		aphids	potato	
PVS (Potato virus S)		aphids	black nightshade, potato, tomato	
**Cheravirus**	**(+)ssRNA**			
AVB (Arracacha virus B)		seed/ pollen	potato	
CRLV (Cherry rasp leaf virus)		nematodes/ seed	tomato	
**Closterovirus**	**(+)ssRNA**			
TV1 (Tobacco virus 1)		not known but other closteroviruses are transmitted by aphids	tobacco	recently described virus
**Comovirus**	**(+)ssRNA**			
APMoV (Andean potato mottle virus)		beetles/contact	eggplant, pepper, potato	
**Crinivirus**	**(+)ssRNA**			
CYSDV (Cucurbit yellow stuning disorder virus)		whitefly *Bemisia tabaci*	potato	only one report, true natural host range on Solanaceae is yet to be discovered
PYVV (Potato yellow vein virus)		whitefly *T. vaporariorum*	black nightshade, potato, tomato	
TICV (Tomato infectious chlorosis virus)		whitefly *T. vaporariorum*	tomato	
ToCV (Tomato chlorosis virus)		whiteflies	tobacco, pepper, tomato, potato, jimson weed, ground cherry, cape gooseberry, tomatillo, eggplant, African eggplant	moderate host range, relatively long latent period in infected host plants
**Cucumovirus**	**(+)ssRNA**			
CMV (Cucumber mosaic virus)		aphids	almost all	extremely broad host range, infecting plants in 85 families and more than 1000 species experimentally
PSV (Peanut stunt virus)		aphids	tobacco	Solanaceous hosts mentions in VIDE database
TAV (Tomato aspermy virus)		aphids	tomato, pepper, petunia	
**Curtovirus**	**ssDNA**			
BCTV (Beet curly top virus)		leafhoppers	pepper, tomato, potato	large host range, widespread
**Deltapartitivirus**	**dsRNA**			
PCV1 (Pepper cryptic virus 1)		pollen/seed	pepper	
PCV2 (Pepper cryptic virus 2)		pollen/seed	pepper	recently described virus
**Elaviroid**	**viroid**			
ELVd (Eggplant latent viroid)		seed	eggplant	
**Fabavirus**	**(+)ssRNA**			
BBWV (Broad bean wilt virus)		aphids	eggplant, petunia, pepper	large host range, now known as two separate species BBWV-1 and BBWV-2
**Ilarvirus**	**(+)ssRNA**			
PMoV (Parietaria mottle virus)		thrips /pollen	pepper, tomato	
PYV (Potato yellowing virus)–tentative member		aphids	potato, pepper	
SnIV 1 (Solanum nigrum ilavirus 1- tentative name)		thrips /pollen	black nightshade, tomato	recently described virus
TomNSV (Tomato necrotic streak virus)		thrips/pollen	tomato	recently described virus
ToNSV (Tomato necrotic spot virus)–tentative member		thrips /pollen	tomato, tobacco, jimson weed	recently described virus
TSV (Tobacco streak virus)		thrips/pollen/seed	potato, tobacco, tomato, jimson weed, petunia, ground cherry	a large host range
**Ipomovirus**	**(+)ssRNA**			
TMMoV (Tomato mild mottle virus)		whitefly *Bemisia tabaci*	tomato, eggplant	
**Macluravirus**	**(+)ssRNA**			
ArLV (Artichoke latent virus)		aphids	petunia	
**Mastrevirus**	**ssDNA**			
CpCDV (Chickpea chlorotic dwarf virus)		leafhoppers *O. orientalis* and *O. albicinctus*	tomato, pepper	contrary to the majority of masterviruses, CpCDV can infect *Solanaceae* hosts
TbYDV (Tobacco yellow dwarf virus)		leafhoppers *Orosius argentatus, O. orientalis,, Anzygina zealandica*	tobacco	
**Nepovirus**	**(+)ssRNA**			
ArMV (Arabis mosaic virus)		nematode vectors, *Xiphinema diversicaudatum, X. coxi*	tomato, petunia, potato, black nightshade, tamarillo, cape gooseberry	
AYRSV (Artichoke yellow ringspot virus)		likely nematodes	tobacco	
CLRV (Cherry leaf roll virus)		nematodes *X. coxi, X. diversicaudatum, X. vuittenezi*	petunia, wild potato	
PBRSV (Potato black ringspot virus)		contact/seed	potato	
PRMV (Peach rosette mosaic virus)		nematodes *Xiphinema americanum, Longidorus diadecturus*	carolina horsenettle	Solanaceous hosts mentions in VIDE database
PVB (Potato virus B)		nematodes *Longidorus spp*.	potato	recently described virus
PVU (Potato virus U)		nematodes *Longidorus spp*./seed	potato	
TBRV (Tomato black ring virus)		nematodes *Longidorus elongatus* and *L. attenuatus*	petunia, potato, tomato	large host range
ToRSV (Tomato ringspot virus)		nematodes *Xiphinema americanum, X. bricolensis, X. californicum, X. rivesi*	tobacco, tomato, petunia, eggplant, pepper, tamarillo	large host range
TRSV (Tobacco ringspot virus)		nematodes *Xiphinema americanum, Longidorus* or *Paralongidorus spp.*	petunia, eggplant, tobacco, tomato	
**Orthotospovirus**	**(+/−) ssRNA**			
TSWV (Tomato spotted wilt orthotospovirus), IYSV (Iris yellow spot orthotospovirus), CaCV (Capsicum chlorosis orthotospovirus), GBNV (Groundnut bud necrosis orthotospovirus), GRSV (Groundnut ringspot orthotospovirus), TCSV (Tomato chlorotic spot orthotospovirus), WBNV (Watermelon bud necrosis orthotospovirus), INSV (Impatiens necrotic spot orthotospovirus) and tentative members PNSV (Pepper necrotic spot virus), TZSV (Tomato zonate spot virus), TNRV (Tomato necrotic ringspot virus), TYRV (Tomato yellow ring virus)		Orthotospoviruses are transmitted by at least 13 thrip species	type species TSWV alone can naturally infect eggplant, potato, tobacco, peper, tomato, black nightshade, petunia, cape gooseberry, tomatillo, Brugmansia, bittersweet, tamarillo, jimson weed …	type species TSWV are widespread and has a very wide host range, other orthotospoviruses infect less plant species, but seem able to naturally infect *Solanaceae*: tomato & pepper (11 viruses), potato (7 viruses)
**Petuvirus**	**dsDNA**			
PVCV (Petunia vein clearing virus)		unknown	petunia	
**Polerovirus**	**(+)ssRNA**			
BWYV (Beet western yellows virus)		aphids	pepper, potato, black nightshade	large host range, probably widespread
PeVYV-1 (Pepper vein yellows virus 1)		aphids	tobacco, pepper	recognized as 6 species PeVYV-1 to -6; recently described viruses
PLRV (Potato leafroll virus)		aphids	potato, tomato, jimson weed, black nightshade, tamarillo	
TVDV (Tobacco vein distorting virus)		aphids	tobacco	the virus can help the vector transmission of TMoV and TBTV (umbraviruses)
**Pomovirus**	**(+)ssRNA**			
CPSbV (Colombian potato soil-borne virus)		likely a soil-borne fungal vector	potato	recently described virus
PMTV (Potato mop-top virus)		Plasmodiophorales	potato	
**Pospiviroid**	**viroid**			
PSTVd (Potato spindle tuber viroid), TASVd (Tomato apical stunt viroid), CEVd (Citrus exocortis viroid), CSVd (Chrysanthemum stunt viroid), TPMVd (Tomato planta macho viroid), TCDVd (Tomato chlorotic dwarf viroid), PCFVd (Pepper chat fruit viroid), CLVd (Columnea latent viroid)		PSTVd alone can spread mechanically, by pollen, seed, aphids, grasshoppers, flea beetles and true bugs	PSTVd alone naturally infects ground cherry, petunia, black nightshade, potato, tomato, pepper, cape gooseberry, jimson weed and brugmansia. Most pospviroids naturally infect Solanaceous plants	widespread, large host range
**Potexvirus**	**(+)ssRNA**			
PAMV (Potato aucuba mosaic virus)		aphids	potato, tamarillo	requiring a helper virus for vector transmission, such as PVY or PVA
PepMV (Pepino mosaic virus)		contact, seeds	eggplant, tobacco, tomato, black nightshade, sweet pepino, potato	transmitted by bumblebees experimentally
PVX (potato virus X)		unknown	cape gooseberry, eggplant, potato, tomato	known for its role in mixed infections with other potato viruses
**Potyvirus**	**(+)ssRNA**			
AEMV (African eggplant mosaic virus)		aphids	African eggplant	recently described virus
BruMV (Brugmansia mosaic virus)		aphids	Brugmansia	recently described virus
BsMoV (Brugmansia suaveolens mottle virus)		aphids	Brugmansia	
CDV (Colombian datura virus)		aphids	jimson weed, petunia, cape gooseberry, sweet pepino, Brugmansia	
ChiVMV (Chilli veinal mottle virus)		aphids	tobacco, pepper, tomato, jimson weed, African eggplant	
DSSV (Datura shoestring virus)		aphids	jimson weed	
HMV (henbane mosaic virus)		aphids	henbane, tomato, tobacco, jimson weed	
PepMoV (Pepper mottle virus)		aphids	tomato, pepper, ground cherry	
PepSMV (Pepper severe mosaic virus)		aphids	pepper	
PepYMV (Pepper yellow mosaic virus)		aphids	tomato, pepper	
PTV (Peru tomato mosaic virus)		aphids	cape gooseberry, tomato, black nightshade, tamarillo	
PVA (potato virus A)		aphids	restricted to Solanaceae	
PVMV (Pepper veinal mottle virus)		aphids	tomato, tobacco, eggplant, black nightshade, jimson weed, ground cherry	
PVV (Potato virus V)		aphids	potato, tomato, tamarillo	
PVY (potato virus Y)		aphids	petunia, cape gooseberry, eggplant, jimson weed, black nightshade, tobacco, potato, tomato, pepper, tamarillo	
TEV (tobacco etch virus)		aphids	wide host range, pepper, tomato, jimson weed, physalis, tobacco, petunia	
TLMV (Tamarillo leaf malformation virus)		aphids	tamarillo	recently described virus
TNSV (Tomato necrotic stunt virus)		aphids	tomato	recently described virus
TVBMV (Tobacco vein banding mosaic virus)		aphids	jimson weed, potato, tobacco	
TVMV (Tobacco vein mottling virus)		aphids	tobacco	
WPMV (Wild potato mosaic virus)		aphids	wild potato	
**Sobemovirus**	**(+)ssRNA**			
SNMoV (Solanum nodiflorum mottle virus)		beetles	*Solanum nordiflorum*	
VTMoV (Velvet tobacco mottle virus)		mirid *Cyrtopeltis nicotianae*	velvet tobacco	
**Solendovirus**	**dsDNA**			
TVCV (Tobacco vein clearing virus)		seed	tobacco	
**Soymovirus**	**dsDNA**			
CmYLCV (Cestrum yellow leaf curling virus)		unknown	*Cestrum* spp.	
**Tepovirus**	**(+)ssRNA**			
PVT (Potato virus T)		seed/pollen	potato	
**Tombusvirus**	**(+)ssRNA**			
EMCV (Eggplant mottled crinkle virus)		unknown	eggplant	
MPV (Moroccan pepper virus)		unknown	tomato, jimson weed, pepper	
PetAMV (Petunia asteroid mosaic virus)		unknown	petunia	
TBSV (Tomato bushy stunt virus)		seed	eggplant, pepper, tomato	
**Tobamovirus**	**(+)ssRNA**			
BrMMV (Brugmansia mild mottle virus)		contact	Brugmansia	
ObPV (Obuda pepper virus)		contact	pepper, tobacco	
PaMMV (Paprika mild mottle virus)		contact	pepper	
PMMoV (Pepper mild mottle virus)		contact/seed	pepper, tobacco, jimson weed, petunia, physalis	widespread
RMV (Ribgrass mosaic virus)		contact	tobacco	
TLV (Tobacco latent virus)		contact/seed	tobacco	
TMGMV (Tobacco mild green mosaic virus)			tomato, pepper, tobacco, petunia	widespread
TMV (Tobacco mosaic virus)		contact/seed	petunia, cape gooseberry, eggplant, black nightshade, tobacco, tomato, potato, pepper	helper virus for satellite tobacco mosaic virus (STNV)
ToBRFV (Tomato brown rugose fruit virus)		contact	tomato	recently described virus
ToMMV (Tomato mottle mosaic)		contact	pepper, tomato	recently described virus
ToMV (Tomato mosaic virus)		contact/seed	petunia, eggplant, potato, pepper, tomato, tamarillo	
YTMMV (Yellow tailflower mild mottle virus)		contact/	yellow tailflower	recently described virus
**Tobravirus**	**(+)ssRNA**			
PepRSV (Pepper ringspot virus)		nematodes *Paratrichodorus minor*	tomato, pepper	
TRV (Tobacco rattle virus)		Trichodorid nematodes	potato, tobacco, pepper	
**Topocuvirus**	**ssDNA**			
TPCTV (Tomato pseudo-curly top virus)		treehopper *Micrutalis malleifera*	tomato	
**Torradovirus**	**(+)ssRNA**			
ToChSV (Tomato chocolate spot virus)		whitefly *Trialeurodes vaporariorum*	tomato	
ToMarV (Tomato marchitez virus)		whiteflies *Trialeurodes abutilonea, T. vaporariorum, and Bemisia tabaci*	tomato, pepper	
ToTV (tomato torrado virus)		whiteflies	tomato, black nightshade	widespread
**Tymovirus**	**(+)ssRNA**			
APLV (Andean potato latent virus)		beetles *Epitrix spp*	potato	
APMMV (Andean potato mild mosaic virus)		beetles	potato	
BeMV (Belladonna mottle virus)		beetle *Epitrix atropae*	ground cherry, deadly nightshade	
DuMV (Dulcamara mottle virus)		beetle *Psylloides affinis*	bittersweet nightshade	
EMV (Eggplant mosaic virus)		beetles	tobacco, tomato, eggplant	
OkMV (Okra mosaic virus)		beetles	ground cherry	
PetVBV (Petunia vein banding virus)		beetles	petunia	
PhyMV (Physalis mottle virus)		beetle *Epitrix cucumeris*	tomatillo	
ToBMV (Tomato blistering mosaic virus)		beetles	tobacco, tomato	recently described virus
**Umbravirus**	**(+)ssRNA**			
TBTV (Tobacco bushy top virus)		aphid	tobacco, tomato, pepper	transmissible by aphids only in presence of TVDV
TMoV (Tabacco mottle virus)		aphids	tobacco	transmissible by aphids only in presence of TVDV
**Unclassified viruses**				
(TNDV) Tobacco necrotic dwarf virus	(+)ssRNA	aphids	tobacco	member of the family Luteoviridae
